# DNA Sequence Profiles of the Colorectal Cancer Critical Gene Set *KRAS-BRAF-PIK3CA-PTEN-TP53* Related to Age at Disease Onset

**DOI:** 10.1371/journal.pone.0013978

**Published:** 2010-11-12

**Authors:** Marianne Berg, Stine A. Danielsen, Terje Ahlquist, Marianne A. Merok, Trude H. Ågesen, Morten H. Vatn, Tom Mala, Ole H. Sjo, Arne Bakka, Ingvild Moberg, Torunn Fetveit, Øystein Mathisen, Anders Husby, Oddvar Sandvik, Arild Nesbakken, Espen Thiis-Evensen, Ragnhild A. Lothe

**Affiliations:** 1 Department of Cancer Prevention, Institute for Cancer Research, Oslo University Hospital, The Norwegian Radium Hospital, Oslo, Norway; 2 Centre for Cancer Biomedicine, University of Oslo, Oslo, Norway; 3 Department of Gastrointestinal Surgery, Oslo University Hospital, Aker, Oslo, Norway; 4 Department of Organ Transplantation, Gastroenterology and Nephrology, Oslo University Hospital, Rikshospitalet, Oslo, Norway; 5 Epigen, Akershus University Hospital, Lørenskog, Norway; 6 Department of Digestive Surgery, Akershus University Hospital, Lørenskog, Norway; 7 Faculty of Medicine, University of Oslo, Oslo, Norway; 8 Department of Surgery, Sørlandet Hospital, Arendal, Norway; 9 Department of Liver, Gastrointestinal and Pediatric Surgery, Oslo University Hospital, Rikshospitalet, Oslo, Norway; 10 Department of Surgery, Diakonhjemmet Hospital, Oslo, Norway; 11 Department of Gastrointestinal Surgery, Sørlandet Hospital, Kristiansand, Norway; The University of Hong Kong, China

## Abstract

The incidence of colorectal cancer (CRC) increases with age and early onset indicates an increased likelihood for genetic predisposition for this disease. The somatic genetics of tumor development in relation to patient age remains mostly unknown. We have examined the mutation status of five known cancer critical genes in relation to age at diagnosis, and compared the genomic complexity of tumors from young patients without known CRC syndromes with those from elderly patients. Among 181 CRC patients, stratified by microsatellite instability status, DNA sequence changes were identified in *KRAS* (32%), *BRAF* (16%), *PIK3CA* (4%), *PTEN* (14%) and *TP53* (51%). In patients younger than 50 years (n = 45), *PIK3CA* mutations were not observed and *TP53* mutations were more frequent than in the older age groups. The total gene mutation index was lowest in tumors from the youngest patients. In contrast, the genome complexity, assessed as copy number aberrations, was highest in tumors from the youngest patients. A comparable number of tumors from young (<50 years) and old patients (>70 years) was quadruple negative for the four predictive gene markers (*KRAS-BRAF-PIK3CA-PTEN*); however, 16% of young versus only 1% of the old patients had tumor mutations in *PTEN/PIK3CA* exclusively. This implies that mutation testing for prediction of EGFR treatment response may be restricted to *KRAS* and *BRAF* in elderly (>70 years) patients. Distinct genetic differences found in tumors from young and elderly patients, whom are comparable for known clinical and pathological variables, indicate that young patients have a different genetic risk profile for CRC development than older patients.

## Introduction

The incidence of colorectal cancer (CRC, MIM#114500) has increased in the western world including Norway during the last 50 years [1]. CRC is typically found in elderly people with a median age at onset of 70 years, and only about five percent of all cases are diagnosed in patients younger than 50 years of age. Hereditary syndromes such as familial adenomatous polyposis (FAP, MIM#175100) and Lynch syndrome (HNPCC, MIM#120435) are found in less than five percent of all CRCs [2]. Although early onset of disease is generally accepted to be indicative of a potential genetic risk, most young at onset cases are regarded as sporadic as no known genetic predisposition is found [3]. Few studies have focused on the somatic tumor development in young patients with no known inherited syndromes [4]–[6].

Receptor tyrosine kinase (RTK) signaling is essential for maintaining the metabolism, proliferation, survival and motility of a cell [7]. Thus, errors in components regulated by receptor tyrosine kinases (RTKs) are commonly observed in human cancers [8], [9]. The oncogenes *KRAS* (HGNC:6407), *BRAF* (HGNC:1097), and *PIK3CA* (HGNC:8975) and the tumor suppressor gene *PTEN* (HGNC:9588) are all affected in response to cytokines, growth factors and hormones signaling through RTK.

Both *KRAS* and *BRAF* have been shown to have activating mutations in ∼70% of CRCs [10], leading to autonomous ERK-signaling [11], [12]. Mutations in components in the PI3-kinase pathway have been reported as mutated in ∼40% in CRC [13], and analyses of the genomic landscape of CRC tumors have shown the PI3K pathway to be affected in a statistically significantly manner [14]. In two of these components, *PTEN* and *PIK3CA*, mutations result in constitutive activation of the PI3K pathway by accumulation of phosphatidylinositol (3,4,5) triphosphate (PIP_3_), which then catalyzes the phosphorylation of AKT. The activated serine-threonine kinase AKT regulates a broad range of target proteins involved in a variety of downstream signaling pathways [15]. Among AKTs downstream targets is the tumor suppressor gene *TP53* (HGNC:11998), a transcription factor that integrates information from many different types of cellular stress, and execute downstream responses appropriate for the given input [16]. *TP53* is found mutated in about half of all colorectal carcinomas [17].

MSI status in the primary tumor has prognostic impact in CRC patients [18]–[20]. *KRAS* and *TP53* status has been associated with clinical end points, the former only with weak association in larger series and the latter only if subgroups of mutations are considered [21]–[23]. Recently, one study has shown that *PIK3CA* mutations may carry prognostic information in tumor stages I–III [24]. This type of information has not been published for *PTEN* mutations.

The metastatic disease treatment targeting EGFR is found to be efficient only if *KRAS* and *BRAF* are not mutated [25]. However, even among the patients with wild type *KRAS* and *BRAF* not all respond to this therapy [26,26]. Lately, it has been proposed that this may be due to mutations in *PIK3CA* and *PTEN*, i.e. only quadruple mutation negative tumors will respond to treatment [26]–[29].

In the present study we have compared the DNA sequence mutation status of the genes *KRAS*, *BRAF*, *PIK3CA, PTEN*, and *TP53* in tumor samples from patients younger than 50 years at diagnosis and without known hereditary syndromes, and stratified according to MSI status and elderly CRC patients.

## Results

Clinicopathological data for the whole series (n = 181), divided into three age groups, are presented in Table 1.

**Table 1:** Clinicopathological features and gene mutation status of colorectal carcinomas for each of the three age groups.

		<50 years, n = 45 (%)	51–70 years, n = 67 (%)	>70 years, n = 69 (%)
**Age at onset of disease**	Min	27	51	71
	Max	50	70	93
	Mean	41	61	80
**Sex**	Female	23 (51)	31 (46)	36 (52)
	Male	22 (49)	36 (54)	33 (48)
**Tumor location in the large bowel**	Right	12 (27)	29 (43)	33 (48)
	Left	16 (36)	15 (22)	20 (29)
	Rectum	17 (38)	23 (34)	16 (23)
**Stage**	I	5 (11)	14 (21)	16 (23)
	II	16 (36)	22 (33)	28 (41)
	III	17 (38)	22 (33)	22 (32)
	IV	7 (16)	9 (13)	3 (4)
**Mutations** §	*KRAS*	13 (30)	20 (30)	24 (35)
	*BRAF*	3 (7)	12 (18)	13 (19)
	*PIK3CA*	0	5 (8)	3 (4)
	*PTEN* *	8 (18)	7 (10)	11 (16)
	*TP53*	29 (64)	26 (39)	38 (55)
	MSI	6 (13)	13 (19)	14 (20)

*Impaired PTEN: Combined results from sequencing and MLPA.

§ Percentages are calculated based on the number of patients with successful analyses.

### Microsatellite instability- and gene mutation status

**Figure 1:**
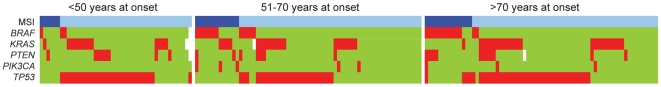
Mutation pattern within age groups. Distribution of mutations in the gene set *KRAS-BRAF-PIK3CA-PTEN-TP53*, and MSI status, within each age group. Color coding: dark blue  =  MSI, light blue  =  MSS, red  =  mutation, green  =  wild type, white  =  not detected.

The MSI- and mutation-frequencies of the analyzed genes are presented in Table 1, and a heat map representing mutation data for all samples is shown in Figure 1. In the total series, MSI was found in 18% of the samples, ranging from 13% in the <50 age group to 20% in the >70 age group. MSI was significantly associated with localized disease, Table S1. The genes were mutated within the expected range of frequencies. Mutation frequencies for each gene independently, and frequencies of combinations of mutations in the genes tested, were equally distributed between all stages. Differences attributed to age at onset are pointed out below.

In total, nine different mutations were observed in *KRAS*, affecting codons 12, 13 and 61. Twenty-eight tumors had the *BRAF* V600E change. Eight *PIK3CA* mutations were identified in exons nine and twenty. Fourteen and fifteen tumors had *PTEN* mutations and deletions, respectively. Ninety-three tumors had *TP53* mutations. Details of all mutations observed, can be found in Table S2.

### Comparison of age groups

The relationships between the clinical and mutational parameters within each age group are given in Table S1. For all age groups, MSI-tumors were most frequently present in right-sided tumor localization. In the oldest patient group MSI and *BRAF* mutations were statistically significantly correlated (*P*<0.001). No such correlation was found in the <50 age group. The MSI tumors within the <50 age group, the 51–70 age group and the >70 age group had 17%, 54% and 79% *BRAF* mutations, respectively (*P* = 0.04). Furthermore, among the six MSI tumors in the <50 group only half of the samples carried mutations in the genes analyzed, whereas among MSI tumors in the other two age groups, all samples displayed one or several mutations.

*KRAS* mutations were found in 57 (32%) tumors in the total series. The mutation frequencies were slightly different between the age groups, Table 1. Most of the mutations were found in codons 12 (68%) and 13 (28%) independent of age group. The codon 61 mutations were only found in samples from the >70 age group.

*KRAS* and *BRAF* mutations were mutually exclusive in the total series. The frequency of tumor mutations in either *KRAS* or *BRAF* increased with patient age, both when comparing age groups and with age as a continuous variable.

Surprisingly, none of the tumors in the <50 age group had *PIK3CA* mutations, whereas five (7%) and three (4%) were found in the 51–70 group and >70 group, respectively.

Eighteen *PTEN* mutations were identified in seven of nine exons (no mutations were observed in exon 4 and 9). The mutations were observed in all age groups, as was the case for *PTEN* deletions. Deletions detected by MLPA were found in eight percent of the samples in total, and coexistence of deletion and alterations detected by sequencing were found in three tumors. Overall, 14% of the tumors had indication of impaired *PTEN* function. The frequency of *PTEN* aberrations showed no statistically significant difference between the age groups; 18%, 10% and 16% in <50 group, 51–70 group and >70 group, respectively.

The number of *TP53* mutated tumors differed among the three age groups (*P* = 0.02). This difference was also significant when analyzing MSS tumors only, (*P = *0.03). Mutations were most prominent in the DNA binding domain, encoded by exons 5–8, and only about five percent of the mutations were observed in exon 4 and exon 11. Moreover, in the <50 age group, *TP53* mutations and MSI were mutually exclusive, and inversely correlated in the 51–70 and >70 groups, respectively.

### Genome complexity and sequence mutations

For a subset of the samples (n = 41) a high resolution dataset of copy number changes obtained by array comparative genome hybridization data (aCGH) was available (Roche NimbleGen, 385 000 oligo probe array) [30]. As previously published [30], we found the <50 group to have a significantly greater number of aberrations than the >70 group, even though the total portion of the genome with aberrations was similar in the two groups, Table 2. A difference in complexity was also found at the gene level, assessed as mutation index: in the older group each patient had 1.5 mutations on average, compared to 1.0 in the younger age group. The distribution of tumor stages was somewhat skewed between the two groups. However, neither the percentage of genomic aberrations, nor number of mutations differed among the tumor stages.

**Table 2:** Gene mutation index and genomic complexity in young and elderly CRC patients.

	Onset <50 years, n = 24	Onset >70 years, n = 17	*P*-values
**Mean number of mutations per patient**	1.04	1.47	0.14
**Median number of regions with copy number aberration per patient**	43.29	26.88	0.02
**Median percentage of normal copy number per patient**	71.72	77.23	0.66

Median number of mutations per patient ( = gene mutation index) is based on data from analyses of *KRAS*, *BRAF*, *PIK3CA, PTEN* and *TP53*. Median number of regions with copy number aberrations ( = genomic complexity) and median percentage of normal copy number per patient are based on results from aCGH analysis.

### Clinical associations

No difference in survival was found comparing the age groups, even when adjusting for tumor stage at diagnosis. There was a trend that the proportion of rectal tumors decreased with increasing age (*P* = 0.08), and the younger patients tended to have a more advanced tumor stage than elderly patients. The prevalence of stage III–IV tumors was 54%, 46%, and 36% in the <50, 51–70 and >70 groups, respectively.

In the total sample set, tumor stage was the most powerful prognostic variable with regard to three-year overall survival, (*P*<0.001). In univariate analysis, patients with *TP53* mutated tumors had poorer survival rates than patients with wild-type *TP53*, 938±31 days vs. 1016±23 days (*P* = 0.04), respectively. However this difference was not significant when correcting for tumor stage. *TP53* mutations were of higher prognostic significance in right-sided tumors, 1051±26 days vs. 883± 65 days for wild type and mutated *TP53*, respectively (*P* = 0.005).

Among patients in the youngest age group, those with *KRAS* mutation had significantly shorter survival than patients with *KRAS* wild-type samples, 841±101 days vs. 1033±35 days (*P* = 0.02), respectively. None of the other mutational parameters showed any statistically significant difference with regard to survival, neither within nor between age groups.

## Discussion

We found that the mutation status of a known cancer gene set varies in CRC patients depending on age at onset of disease, and provide further evidence of differences in the somatic development of tumors in young and elderly patients.

### Microsatellite instability status

In all age groups, MSI was statistically significantly associated with right-sided tumors, as expected [18], [18], [31,32]. The frequency of MSI in the present series was somewhat higher than reported elsewhere [33], [34], which is best explained by a random excess of right-sided tumors in the total series. However, for the group of patients younger than 50 years at diagnosis the frequency observed were slightly lower than expected [33], which partly could be explained by the exclusion of patients with known CRC syndromes in this study.

In patients younger than 50 years only 50% of the MSI samples were mutated in one or more of the genes analyzed, whereas in patients older than 50 all MSI samples displayed gene alterations. Additionally, *BRAF* mutations were not statistically significantly associated with MSI in the <50 years group, contrary to what was expected [35]. The mutation in *BRAF* ^V600E^ is observed to induce down-regulation of essential DNA repair genes [36], with potential to induce MSI in the cell, explaining this correlation. As *BRAF* mutations are not observed in tumors from patients with Lynch syndrome [37], we cannot exclude that MSI/*BRAF* wild-type samples in the present series are from clinically undetected Lynch syndrome patients.

### Gene mutations

Mutations frequencies in *KRAS, BRAF, PIK3CA, PTEN*, and *TP53* were found to be within previously reported range supporting the representativeness of the series [38].

The coexistence of *BRAF* and *KRAS* mutations is presumed to be incompatible with proliferation, hence a negative selection for concurrent existence of both mutations [12]. We did confirm an inverse correlation between mutations of these two genes [39]. In addition, an increasing number of tumors with either *KRAS or BRAF* mutation was observed with increasing age, in agreement with a recent report on young patients with colorectal cancer [6].

In the total series, tumors displaying *BRAF* ^V600E^ were typically *TP53* wild type and *PTEN* mutated (Table S1), which was also the case for tumors within the >70 age group. Thus, “*BRAF* ^V600E^, *TP53*wt, *PTEN*mut” may be regarded as a feature of genuine sporadic tumors.

In comparison to the reported 14% frequency of *PIK3CA* mutations in colorectal carcinomas in the COSMIC database [38], the current series shows only 4% mutations. This may partly be explained by the fact that the current series is enriched with samples from young patients, and their tumors did not have *PIK3CA* mutations. However, this latter observation is in contrast with two previous studies [6], [40] that reports two/one patients younger than 50 years with *PIK3CA* mutations. The different methodologies used, sample type, formalin-fixed or fresh frozen tissue, and the size of the series may partly explain this difference in mutation frequency among the studies. Mutation in either the oncogene *PIK3CA* or the tumor suppressor gene *PTEN* may lead to an accumulation of PIP_3_, and downstream AKT activation. However, since a sequence change in *PTEN* imply one remaining wild type allele, additional activating mutation in the same pathway may be beneficial for the tumor cells [41]. We found 32 tumors with alteration(s) in each of *PIK3CA* and/or *PTEN*, including seven tumors with several *PTEN* aberrations. The whole coding sequence of *PTEN* has been explored [42]–[48], but none of the studies have investigated tumors from young patients specifically. To our knowledge this is the first report combining *PTEN* whole coding sequence data with the gross deletion pattern in CRC from different age groups.

The *TP53* mutation frequency was significantly higher in the <50 age group, than in the other age groups, which is best explained by the higher frequency of left sided and rectal cancers in the <50 age group. As expected, *TP53* mutations were associated with MSS tumors, and the significant difference between the age groups was still valid when analyzing MSS tumors only.

A mutation index was calculated and tumors from elderly patients were found to have accumulated more gene sequence changes than the ones from young patients (1.5 vs. 1.0) which might indicate a time difference in tumor development.

### Distinct genetic make-up of tumors from young and elderly patients

From aCGH data on a subset of 41 samples, we observed that patients younger than 50 years had significantly more aberrations in their tumors than patients >70 years did, *i.e*. a higher degree of genomic complexity. Contrary, as mentioned above, the tumors from elderly patients had the highest gene mutation index, even though this group included fewer advanced stages. Taken together, one may speculate that among the young patients there are carriers with genetic predisposition affecting genes encoding proteins that ensure correct chromosome segregation during mitosis, rather than specific pathways.

Bardelli *et al.* [29] reported that patients with metastatic disease that are “quadruple negative” in the *KRAS-BRAF-PIK3CA-PTEN* gene set have the highest probability of response to anti-EGFR therapies. However, currently only *KRAS* testing is recommended prior to decision with regard to such treatment, and *BRAF* testing is optional [49].

**Figure 2:**
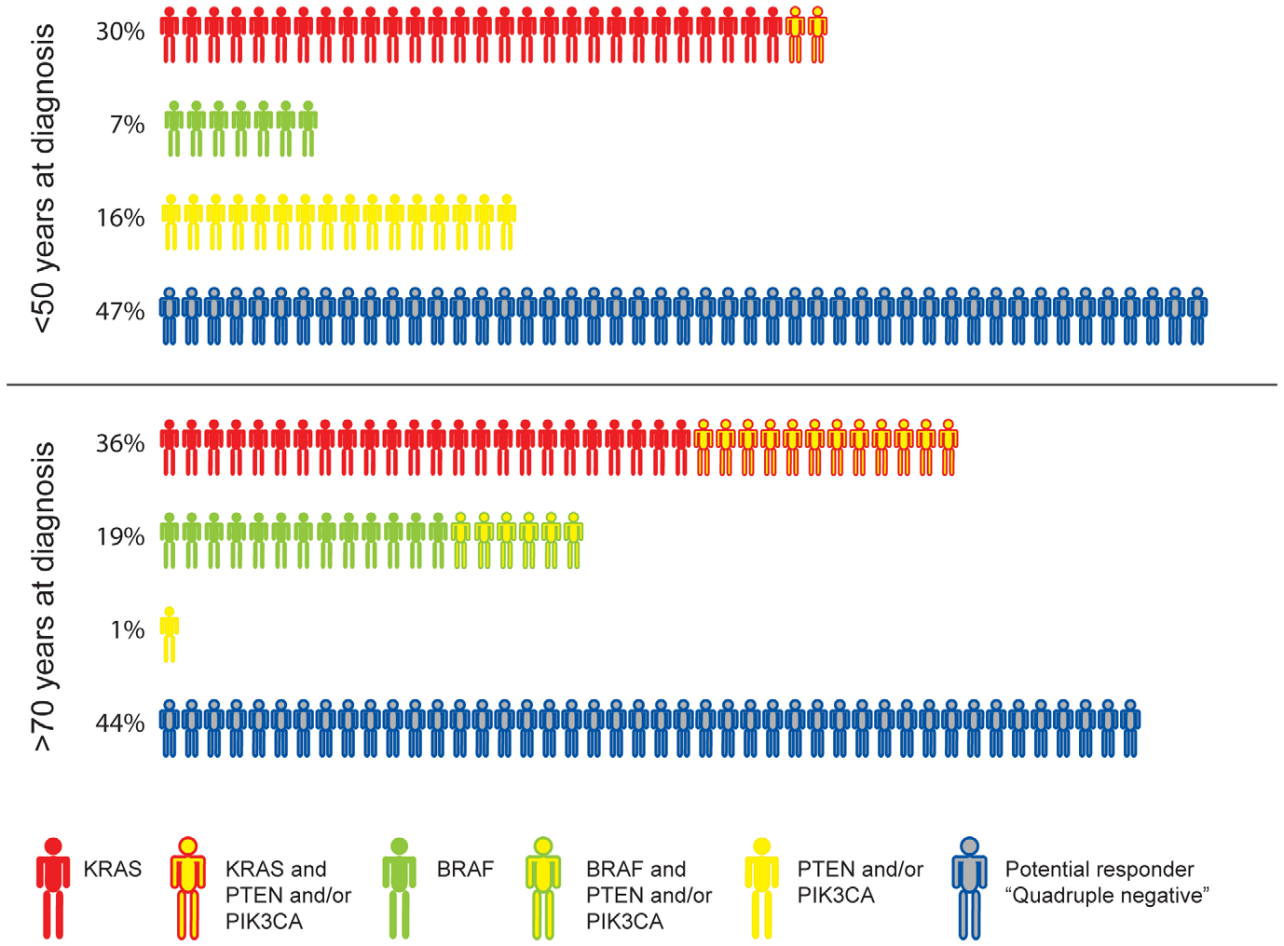
Graphic representation of patients with hypothesized response to EGFR-targeted therapy. Percent-wise distribution of aberrations for *KRAS*, *BRAF*, *PIK3CA* and *PTEN* and their combinations, in the <50 age group and >70 age group. Patients negative for all mutations (quadruple negative) are potential responders to anti-EGFR-therapy. (Modified from Bardelli *et al.* [*29*])

In the present series of primary cancers, mutation frequencies in these four genes were equally distributed between all tumor stages. Therefore, we have in the following hypothesized the number of potential responders based on the whole dataset (Figure 2). When considering tumors with impaired *PTEN* and/or mutated *PIK3CA* exclusively, the youngest age group displayed 16% (n = 7) mutations, compared to 1.4% (n = 1) in the oldest age group. This implies that for older patients, *KRAS* and *BRAF* mutation status alone will reveal 98% of the patients unsuitable for anti-EGFR therapy, as compared to 71% in patients <50 years. The effect of mutations in *PIK3CA* and *PTEN* are debated with regard to anti-EGFR therapy [26], [28], [29]. Notwithstanding, if their mutational status does not influence on the effect of anti-EGFR treatment, the younger patients will be expected to have an even larger proportion of potential responders to this type of treatment than the older patients (Figure 2).

### Association to clinical data

The consecutive sample series of colorectal carcinomas from a single hospital showed overall distribution of clinicopathological data as expected for a Norwegian cohort [1]. The proportion of rectal cancers decreased with increasing age at onset, whereas the opposite trend was seen for right-sided tumors, as reported by others [50], [51]. Furthermore, a larger part of the patients diagnosed before age 50 years had more advanced disease stage compared to the older age groups. This may reflect that CRC is unexpected in young adults and symptoms are neglected both by the patient and the physician [52]. Reports of young colorectal cancer patients differs with regards to aggressiveness of the disease, and hence outcome [3], [52], [53]. However, in the present study, the three-year overall survival was equal in the three age groups, even when adjusting for tumor stage. This might indicate a more aggressive disease in young patients, as older people are expected to have a higher mortality rate. Furthermore, the observed higher degree of genomic complexity in tumors from young versus elderly patients also may be indicative of a difference in aggressiveness. It should be noted, that the young patients included are not carriers of known colorectal cancer syndromes according to clinical criteria only.

The prognostic and predictive value of *TP53* is still controversial [54]. In our dataset patients with *TP53* mutations had significantly shorter survival than wild type patients. Furthermore, for patients with right-sided or MSI tumors *TP53* mutations showed even greater prognostic value, in line with results reported by the *TP53*-CRC collaborative study group [55].

In a recent study, *PIK3CA* mutations were found to serve as surrogate markers for poor survival in colorectal cancer patients [24]. As few *PIK3CA* mutations were observed in our sample set, we could not confirm these results. However, mutations were found only in samples from older patients, and were not present in patients with distant metastases, and only few in stage III patients. Thus, our data suggest that *PIK3CA* mutations are important for tumorigenesis among elderly patients, but do not serve as a marker for the ability to metastasize.

### Conclusion

We have found distinct differences in the genetic make-up of carcinomas from young and elderly patients. The tumors from young patients whom are not carriers of known hereditary CRC syndromes have less gene mutations, but more copy number aberrations across the genome. These data suggest that some young patients may have a potential increased risk for cancer caused by alterations in gene(s) involved in maintaining correct chromosome segregation.

## Materials and Methods

### Ethics statement

Written informed consent was obtained from all subjects included. The research biobanks are registered according to national legislation and the research studies are approved by the Regional Committee for Medical Research Ethics (REK South-East: 1.2005.1629; REK South, 2003: S-02126).

### Patients and tumor samples

A total of 181 patients were included in the study, *i.e.* a consecutive series of 132 patients enriched with a series of 49 patients with disease at young age (<50 years). Each series are described in the following paragraphs. Clinical data were collected prospectively. Death data were retrieved from either the hospital records or from the Norwegian Population Registry. The clinical and pathological data of these two series are shown in Table S3.

#### Consecutive series (n = 132)

Patients undergoing elective resection of colorectal adenocarcinoma in 2005–2007 at Oslo University Hospital, Aker, Oslo, were included. Patients with FAP- or Lynch syndrome were excluded based on clinical criteria. Tumor tissue was sampled immediately after resection of the specimen and instantly frozen in liquid nitrogen. All tissue specimens were evaluated by a pathologist to establish differentiation grade of the carcinoma and tumor cell content.

#### Early onset series (n = 49)

Samples from a biobank from a multi-hospital study (INFAC-study - individuals with familial risk for cancer), including Oslo University Hospital, Aker, Oslo and five other hospitals in the same geographical region of Norway enrolled from 2003–2008, were included in the present study. The inclusion criteria in the INFAC-study were age <55 years, excluding those who fulfilled criteria of all known CRC syndromes. Tumor tissue and corresponding normal mucosa samples were taken from the resected specimen in the operation theatre and promptly incubated in RNAlater. The tissue was transferred to new tubes after 36–72 hours, and stored at minus 80°C until use. DNA from normal and cancerous tissues was extracted, and used for analysis.

#### Samples included in CGH analysis

For a subset of the patients (n = 41) data from aCGH analyses were available. This subset included 24 patients younger than 50 years, and 17 older than 70 years at primary diagnosis. Clinical and mutational data for these patients are presented in Table S2.

#### Grouping of patients according to age

Patients from the early onset series were included to enrich the number of early onset patients in the total sample set. Patients from the early onset series were selected to match patients younger than 55 years in the consecutive series with regard to gender, median age, tumor stage and localization, Table S3. Thereafter, samples from the two series were combined and divided into three age groups; under age 50 years, 51 through 70 years and over 70 years, referred to as <50, 51–70 and >70, respectively. The mean age at primary diagnosis was 70 years in the consecutive series, and that was chosen as cutoff for the oldest age group. The cutoff for the youngest age group was set at 50 years, since early onset may be due to inherited genetic factors, and only ∼5% of all CRC are diagnosed before 50 years of age. Tumors in the colon proximal to the splenic flexure were defined as right-sided: those in the remaining colon were regarded as left-sided whereas rectum was defined as 15 cm from the anal verge when measured with a rigid proctoscope. Localized disease was defined for UICC/AJCC stages I and II and regional disease for stages III and IV.

### Microsatellite instability analysis

For all patients, tumor and corresponding normal tissue or blood was analyzed using the Bethesda markers [56]. The analysis was performed as described by Wu *et al.* [57]. High degree of microsatellite instability (MSI-H) in tumor DNA, as compared to corresponding normal DNA, was defined if two or more markers showed aberrant peak profile after fragment analysis. If both the normal sample and the tumor sample from a patient showed the same pattern for all markers, the tumor was regarded microsatellite stable (MSS).

### Mutation analysis for KRAS, BRAF, PIK3CA, PTEN and TP53

For *BRAF* and *KRAS*, activating mutations at positions known to frequently carry alterations are reported [11], [58]. In the *PIK3CA* gene mutations in colorectal cancers cluster in exons 9 and 20 [59], and for *TP53* and *PTEN* mutations are reported throughout the whole coding sequence [17], [60].

For *TP53* and *PTEN*, the total coding sequence was amplified in multiplex PCR reactions, using multiplex PCR kit as recommended by the vendor (QIAGEN, GmBH, Hilden, Germany) [30], [60]. Singleplex PCR reactions using HotStar Taq (QIAGEN) were used to amplify *PIK3CA*, *KRAS* and *BRAF* amplicons [10], [59], [61]. In *PIK3CA* exons 9 and 20 were amplified, in *KRAS*, exons 2 and 3, including the frequently mutated codons 12, 13 and 61, and in *BRAF* exon 15, including the mutation in codon 600, were amplified. Primers, running conditions and fragment details are described in Table S4.

PCR products were purified using Sephadex columns (Millipore, Billerica, MA, US and GE Healthcare, Chalfont St.Giles, UK) prior to incorporation of dye labeled ddNTP's and sequencing on the 3730 DNA Analyzer (Applied Biosystems, Foster City, CA, US) [30]. In cases where a mutation was detected, a new independent PCR product was subjected to sequencing to confirm the result.

### Multiplex ligation-dependent probe amplification (MLPA) of PTEN

Salsa MLPA kit P225-B2 PTEN (MRC Holland, Amsterdam, Netherlands) were used according to instructions from distributor. In total, 25 probes cover the *PTEN* gene, a minimum of two probes for each of the nine exons. Also, 10 probes located elsewhere on chromosome 10, and additional 12 reference probes located on other chromosomes serves as controls. Amplified fragments were analyzed on a 3730 DNA Analyzer (Applied Biosystems). Raw data were analyzed with Coffalyzer® (MRC Holland) with default settings. Cutoffs of 1.2 and 0.8 were used for scoring of gains and losses, respectively, in addition to individual evaluation of the plot from each sample. This scoring is determined according to the following calculation: one copy gain or loss in 60% of the cells in a triploid tumor equals 1.2 and 0.8, respectively. At least two neighboring probes, located in the same exon, with concomitant gain or loss, were confined for assigning aberrant copy number in a region.

### Statistical analyses

Clinical variables were tested for associations with mutational results for all genes analyzed, and all genes were tested for inter-relational associations. All statistical analyses were performed using the software package PASW 17.0 (SPSS Inc., Chicago, IL, US). Fisher's exact test was used for cross-tabulated variables. Student T-test was performed when calculating the relationship between a continuous variable and a categorical variable. Survival analyses were performed using the Kaplan–Meier life table analyses with three years overall survival, and log rank test used to compare the survival curves. For all statistical tests, a two-tailed *P*<0.05 was considered significant.

## Supporting Information

Table S1 Relation of mutational and clinical data on tumors from included patients. NS = not significant, NA = not applicable. All P-values <0.20 are shown in the table, and P<0.05 is high-lighted in bold.
(0.03 MB XLS)

Table S2 Details of clinical and mutational data from *KRAS*, *BRAF*, *PIK3CA*, *PTEN* and *TP53*. * F = female, M = male. # patients included in Table 2. § results from MLPA.
(0.05 MB XLS)

Table S3 Clinical characteristics in different age groups. Distribution of clinical data in the total consecutive series, young in consecutive series (patients under age 55 years), early onset series (<55 years), and in the total series of patients.
(0.03 MB XLS)

Table S4 PCR and primer details for genes analyzed. *M13 forward primer (TGTAAAACGACGGCCAGT). #M13 reverse primer (CAGGAAACAGCTATGACC).
(0.03 MB XLS)

## References

[1] Cancer Registry of Norway (2009). Cancer in Norway 2008 - Cancer incidence, mortality, survival and prevalence in Norway..

[2] Lynch HT, de la Chapelle A (2003). Hereditary colorectal cancer.. N Engl J Med.

[3] Zbuk K, Sidebotham EL, Bleyer A, La Quaglia MP (2009). Colorectal cancer in young adults.. Semin Oncol.

[4] Liang JT, Huang KC, Cheng AL, Jeng YM, Wu MS (2003). Clinicopathological and molecular biological features of colorectal cancer in patients less than 40 years of age.. Br J Surg.

[5] Khan SA, Idrees K, Forslund A, Zeng Z, Rosenberg S (2008). Genetic variants in germline TP53 and MDM2 SNP309 are not associated with early onset colorectal cancer.. J Surg Oncol.

[6] Yantiss RK, Goodarzi M, Zhou XK, Rennert H, Pirog EC (2009). Clinical, pathologic, and molecular features of early-onset colorectal carcinoma.. Am J Surg Pathol.

[7] Rusten TE, Haglund K, Stenmark H (2007). Aberrant receptor signaling and trafficking as mechanisms in oncogenesis.. Crit Rev Oncog.

[8] Kang S, Bader AG, Vogt PK (2005). Phosphatidylinositol 3-kinase mutations identified in human cancer are oncogenic.. Proc Natl Acad Sci U S A.

[9] Bos JL (1989). ras oncogenes in human cancer: a review.. Cancer Res.

[10] Ahlquist T, Bottillo I, Danielsen SA, Meling GI, Rognum TO (2008). RAS signaling in colorectal carcinomas through alteration of RAS, RAF, NF1, and/or RASSF1A.. Neoplasia.

[11] Wan PT, Garnett MJ, Roe SM, Lee S, Niculescu-Duvaz D (2004). Mechanism of activation of the RAF-ERK signaling pathway by oncogenic mutations of B-RAF.. Cell.

[12] Garnett MJ, Marais R (2004). Guilty as charged: B-RAF is a human oncogene.. Cancer Cell.

[13] Samuels Y, Ericson K (2006). Oncogenic PI3K and its role in cancer.. Curr Opin Oncol.

[14] Wood LD, Parsons DW, Jones S, Lin J, Sjoblom T (2007). The genomic landscapes of human breast and colorectal cancers.. Science.

[15] Ericson K, Gan C, Cheong I, Rago C, Samuels Y (2010). Genetic inactivation of AKT1, AKT2, and PDPK1 in human colorectal cancer cells clarifies their roles in tumor growth regulation.. Proc Natl Acad Sci U S A.

[16] Sengupta S, Harris CC (2005). p53: traffic cop at the crossroads of DNA repair and recombination.. Nat Rev Mol Cell Biol.

[17] Petitjean A, Mathe E, Kato S, Ishioka C, Tavtigian SV (2007). Impact of mutant p53 functional properties on TP53 mutation patterns and tumor phenotype: lessons from recent developments in the IARC TP53 database.. Hum Mutat.

[18] Lothe RA, Peltomaki P, Meling GI, Aaltonen LA, Nystrom-Lahti M (1993). Genomic instability in colorectal cancer: relationship to clinicopathological variables and family history.. Cancer Res.

[19] Popat S, Hubner R, Houlston RS (2005). Systematic review of microsatellite instability and colorectal cancer prognosis.. J Clin Oncol.

[20] Samowitz WS, Curtin K, Wolff RK, Tripp SR, Caan BJ (2009). Microsatellite instability and survival in rectal cancer.. Cancer Causes Control.

[21] Andreyev HJ, Norman AR, Cunningham D, Oates JR, Clarke PA (1998). Kirsten ras mutations in patients with colorectal cancer: the multicenter “RASCAL” study.. J Natl Cancer Inst.

[22] Diep CB, Thorstensen L, Meling GI, Skovlund E, Rognum TO (2003). Genetic tumor markers with prognostic impact in Dukes' stages B and C colorectal cancer patients.. J Clin Oncol.

[23] Petitjean A, Achatz MI, Borresen-Dale AL, Hainaut P, Olivier M (2007). TP53 mutations in human cancers: functional selection and impact on cancer prognosis and outcomes.. Oncogene.

[24] Ogino S, Nosho K, Kirkner GJ, Shima K, Irahara N (2009). PIK3CA mutation is associated with poor prognosis among patients with curatively resected colon cancer.. J Clin Oncol.

[25] Lievre A, Bachet JB, Le CD, Boige V, Landi B (2006). KRAS mutation status is predictive of response to cetuximab therapy in colorectal cancer.. Cancer Res.

[26] Di Nicolantonio F, Martini M, Molinari F, Sartore-Bianchi A, Arena S (2008). Wild-type BRAF is required for response to panitumumab or cetuximab in metastatic colorectal cancer.. J Clin Oncol.

[27] Amado RG, Wolf M, Peeters M, Van CE, Siena S (2008). Wild-type KRAS is required for panitumumab efficacy in patients with metastatic colorectal cancer.. J Clin Oncol.

[28] Tol J, Dijkstra JR, Klomp M, Teerenstra S, Dommerholt M (2010). Markers for EGFR pathway activation as predictor of outcome in metastatic colorectal cancer patients treated with or without cetuximab.. Eur J Cancer.

[29] Bardelli A, Siena S (2010). Molecular mechanisms of resistance to cetuximab and panitumumab in colorectal cancer.. J Clin Oncol.

[30] Berg M, Ågesen T, Thiis-Evensen E, INFAC-study group, Merok MA, et al. (2010). Distinct high resolution genome profiles of early onset and late onset colorectal cancer integrated with gene expression data identify candidate susceptibility loci.. Molecular Cancer.

[31] Thibodeau SN, Bren G, Schaid D (1993). Microsatellite instability in cancer of the proximal colon.. Science.

[32] Boland CR, Goel A (2010). Microsatellite instability in colorectal cancer.. Gastroenterology.

[33] Gryfe R, Kim H, Hsieh ET, Aronson MD, Holowaty EJ (2000). Tumor microsatellite instability and clinical outcome in young patients with colorectal cancer.. N Engl J Med.

[34] Lothe RA (1997). Microsatellite instability in human solid tumors.. Mol Med Today.

[35] Jass JR (2007). Classification of colorectal cancer based on correlation of clinical, morphological and molecular features.. Histopathology.

[36] Oikonomou E, Makrodouli E, Evagelidou M, Joyce T, Probert L (2009). BRAF(V600E) efficient transformation and induction of microsatellite instability versus KRAS(G12V) induction of senescence markers in human colon cancer cells.. Neoplasia.

[37] McGivern A, Wynter CV, Whitehall VL, Kambara T, Spring KJ (2004). Promoter hypermethylation frequency and BRAF mutations distinguish hereditary non-polyposis colon cancer from sporadic MSI-H colon cancer.. Fam Cancer.

[38] Forbes SA, Bhamra G, Bamford S, Dawson E, Kok C (2008). The Catalogue of Somatic Mutations in Cancer (COSMIC).. Curr Protoc Hum Genet Chapter.

[39] Chan TL, Zhao W, Leung SY, Yuen ST (2003). BRAF and KRAS mutations in colorectal hyperplastic polyps and serrated adenomas.. Cancer Res.

[40] Herreros-Villanueva M, Gomez-Manero N, Muniz P, Garcia-Giron C, Coma Del Corral MJ (2010). PIK3CA mutations in KRAS and BRAF wild type colorectal cancer patients. A study of Spanish population.. Mol Biol Rep.

[41] Perrone F, Lampis A, Orsenigo M, Di BM, Gevorgyan A (2009). PI3KCA/PTEN deregulation contributes to impaired responses to cetuximab in metastatic colorectal cancer patients.. Ann Oncol.

[42] Wang ZJ, Taylor F, Churchman M, Norbury G, Tomlinson I (1998). Genetic pathways of colorectal carcinogenesis rarely involve the PTEN and LKB1 genes outside the inherited hamartoma syndromes.. Am J Pathol.

[43] Chang JG, Chen YJ, Perng LI, Wang NM, Kao MC (1999). Mutation analysis of the PTEN/MMAC1 gene in cancers of the digestive tract.. Eur J Cancer.

[44] Dicuonzo G, Angeletti S, Garcia-Foncillas J, Brugarolas A, Okrouzhnov Y (2001). Colorectal carcinomas and PTEN/MMAC1 gene mutations.. Clin Cancer Res.

[45] Shin KH, Park YJ, Park JG (2001). PTEN gene mutations in colorectal cancers displaying microsatellite instability.. Cancer Lett.

[46] Zhou XP, Loukola A, Salovaara R, Nystrom-Lahti M, Peltomaki P (2002). PTEN mutational spectra, expression levels, and subcellular localization in microsatellite stable and unstable colorectal cancers.. Am J Pathol.

[47] Nassif NT, Lobo GP, Wu X, Henderson CJ, Morrison CD (2004). PTEN mutations are common in sporadic microsatellite stable colorectal cancer.. Oncogene.

[48] Karoui M, Tresallet C, Julie C, Zimmermann U, Staroz F (2004). Loss of heterozygosity on 10q and mutational status of PTEN and BMPR1A in colorectal primary tumours and metastases.. Br J Cancer.

[49] Engstrom PF (2008). Systemic therapy for advanced or metastatic colorectal cancer: National Comprehensive Cancer Network guidelines for combining anti-vascular endothelial growth factor and anti-epidermal growth factor receptor monoclonal antibodies with chemotherapy.. Pharmacotherapy.

[50] de Jong UW, Day NE, Muir CS, Barclay TH, Bras G (1972). The distribution of cancer within the large bowel.. Int J Cancer.

[51] Gonzalez EC, Roetzheim RG, Ferrante JM, Campbell R (2001). Predictors of proximal vs. distal colorectal cancers.. Dis Colon Rectum.

[52] Frizis H, Papadopoulos A, Akritidis G, Frizis HR, Hatzitheoharis G (2004). Are there any differences in colorectal cancer between young and elderly patients?. Tech Coloproctol.

[53] O'Connell JB, Maggard MA, Livingston EH, Yo CK (2004). Colorectal cancer in the young.. Am J Surg.

[54] Brosh R, Rotter V (2009). When mutants gain new powers: news from the mutant p53 field.. Nat Rev Cancer.

[55] Russo A, Bazan V, Iacopetta B, Kerr D, Soussi T (2005). The TP53 colorectal cancer international collaborative study on the prognostic and predictive significance of p53 mutation: influence of tumor site, type of mutation, and adjuvant treatment.. J Clin Oncol.

[56] Boland CR, Thibodeau SN, Hamilton SR, Sidransky D, Eshleman JR (1998). A National Cancer Institute Workshop on Microsatellite Instability for cancer detection and familial predisposition: development of international criteria for the determination of microsatellite instability in colorectal cancer.. Cancer Res.

[57] Wu Q, Lothe RA, Ahlquist T, Silins I, Trope CG (2007). DNA methylation profiling of ovarian carcinomas and their in vitro models identifies HOXA9, HOXB5, SCGB3A1, and CRABP1 as novel targets.. Mol Cancer.

[58] Land H, Parada LF, Weinberg RA (1983). Cellular oncogenes and multistep carcinogenesis.. Science.

[59] Samuels Y, Wang Z, Bardelli A, Silliman N, Ptak J (2004). High frequency of mutations of the PIK3CA gene in human cancers.. Science.

[60] Danielsen SA, Lind GE, Bjornslett M, Meling GI, Rognum TO (2008). Novel mutations of the suppressor gene PTEN in colorectal carcinomas stratified by microsatellite instability- and TP53 mutation- status.. Hum Mutat.

[61] Davies H, Bignell GR, Cox C, Stephens P, Edkins S (2002). Mutations of the BRAF gene in human cancer.. Nature.

